# Application of Machine Learning to Ultrasonography in Identifying Anatomical Landmarks for Cricothyroidotomy Among Female Adults: A Multi-center Prospective Observational Study

**DOI:** 10.1007/s10278-023-00929-3

**Published:** 2024-01-10

**Authors:** Chih-Hung Wang, Jia-Da Li, Cheng-Yi Wu, Yu-Chen Wu, Joyce Tay, Meng-Che Wu, Ching-Hang Hsu, Yi-Kuan Liu, Chu-Song Chen, Chien-Hua Huang

**Affiliations:** 1 Department of Emergency Medicine, National Taiwan University Hospital, Taipei, Taiwan; 2 Department of Emergency Medicine, College of Medicine, National Taiwan University, Taipei, Taiwan; 3 NTU Joint Research Center for AI Technology and All Vista Healthcare, National Taiwan University, Taipei, Taiwan; 4 Department of Computer Science and Information Engineering, National Taiwan University, Taipei, Taiwan; 5 Institute of Information Science, Academia Sinica, Taipei, Taiwan

**Keywords:** Cricothyroidotomy, Female, Machine learning, Object detection, Ultrasound

## Abstract

We aimed to develop machine learning (ML)-based algorithms to assist physicians in ultrasound-guided localization of cricoid cartilage (CC) and thyroid cartilage (TC) in cricothyroidotomy. Adult female volunteers were prospectively recruited from two hospitals between September and December, 2020. Ultrasonographic images were collected via a modified longitudinal technique. You Only Look Once (YOLOv5s), Faster Regions with Convolutional Neural Network features (Faster R-CNN), and Single Shot Detector (SSD) were selected as the model architectures. A total of 488 women (mean age: 36.0 years) participated in the study, contributing to a total of 292,053 frames of ultrasonographic images. The derived ML-based algorithms demonstrated excellent discriminative performance for the presence of CC (area under the receiver operating characteristic curve [AUC]: YOLOv5s, 0.989, 95% confidence interval [CI]: 0.982–0.994; Faster R-CNN, 0.986, 95% CI: 0.980–0.991; SSD, 0.968, 95% CI: 0.956–0.977) and TC (AUC: YOLOv5s, 0.989, 95% CI: 0.977–0.997; Faster R-CNN, 0.981, 95% CI: 0.965–0.991; SSD, 0.982, 95% CI: 0.973–0.990). Furthermore, in the frames where the model could correctly indicate the presence of CC or TC, it also accurately localized CC (intersection-over-union: YOLOv5s, 0.753, 95% CI: 0.739–0.765; Faster R-CNN, 0.720, 95% CI: 0.709–0.732; SSD, 0.739, 95% CI: 0.726–0.751) or TC (intersection-over-union: YOLOv5s, 0.739, 95% CI: 0.722–0.755; Faster R-CNN, 0.709, 95% CI: 0.687–0.730; SSD, 0.713, 95% CI: 0.695–0.730). The ML-based algorithms could identify anatomical landmarks for cricothyroidotomy in adult females with favorable discriminative and localization performance. Further studies are warranted to transfer this algorithm to hand-held portable ultrasound devices for clinical use.

**Supplementary Information:**

The online version contains supplementary material available at 10.1007/s10278-023-00929-3.

## Introduction

Managing the difficult airway is challenging. The reported incidence of difficult airway ranges between 2 and 15% [1–6]. By creating direct access to the trachea via the cricothyroid membrane (CTM), emergent cricothyroidotomy is recommended by the Difficult Airway Society guidelines [7] as the last resort for “can’t intubate, can’t oxygenate” scenarios with worsening hypoxia; however, attempts to secure the airway with cricothyroidotomy are unsuccessful in a reported 64% of cases [8].

Precise and quick identification of the CTM is critical for successful cricothyroidotomy. This technique is traditionally taught by using surface landmarks to identify the CTM, which spans the inferior border of the thyroid cartilage (TC) and the superior border of the cricoid cartilage (CC). Reliance on manual palpation may be insufficient for correct identification of the CTM, particularly in women, whose external landmarks are barely palpable. In one study, the CTM was misidentified at manual palpation in 81% of female participants [9].

In a meta-analysis, Hung et al. [10] indicated that compared with manual palpation, applying an ultrasound-guided technique was significantly associated with a lower failure rate in identifying the CTM. Nevertheless, Hung et al. [10] also found that the procedural time for the ultrasound-guided technique tended to be longer than that of the manual palpation method, which is a concern when faced with an immediately life-threatening condition [10].

Even after intensive training, ultrasound-assisted identification of the CTM is not failproof, and a failure rate as high as 26% is reported [10]. Machine learning (ML) has been increasingly used in the field of ultrasonography. Given its potential for achieving quick and accurate object detection, in the current study, we developed an ML-based algorithm to identify the anatomical landmarks of cricothyroidotomy (i.e., the CC and TC) among female adults.

## Materials and Methods

This multi-center prospective observational study was approved by the Research Ethics Committee of the XXX Hospital (XXXH) (reference number: 202006015RIND) and performed in accordance with the ethical standards as laid down in the 1964 Declaration of Helsinki and its later amendments or comparable ethical standards. Written informed consent was obtained from all participants. The study results are reported according to the Checklist for Artificial Intelligence in Medical Imaging (CLAIM) [11].

### Setting and Participants

We recruited volunteer participants from among hospital employees at XXXH (Taipei, Taiwan) and the XXXH Yunlin Branch (XXXH-YB) (Yunlin, Taiwan) from September 1, 2020 to December 31, 2020. Participants were passively recruited using word of mouth. Women aged 20 years and older were eligible for inclusion. Participants were excluded if they met the following exclusion criteria: (1) history of previous neck surgery or radiation; (2) inability to extend their neck actively. All eligible participants provided written informed consent. Because of the observational study design, the number of eligible participants during the enrolment period determined the final sample size.

### Data Collection and Image Acquisition

All participants provided baseline characteristic data, including age, weight, and height. The ultrasonographic assessment was performed while volunteers were in a supine position with the neck extended. In this position, the participants extended their necks maximally while remaining comfortable. The investigator stood on the right-hand side of the participant and used the modified longitudinal technique [12, 13] to collect the ultrasonographic imaging data. As shown in Video A.1, the transducer was placed longitudinally along the midline of the neck, above the suprasternal notch, to produce a sagittal image. From there, the operator slides the transducer cephalad to obtain a sequence of images, including (1) a series of hypoechoic rings (tracheal rings) superficial to the hyperechoic air-tissue border; (2) a cuboid hypoechoic ring (CC), which was larger and more anterior than the tracheal rings; (3) a hyperechoic band that runs between the hypoechoic CC and TC; and (4) a hypoechoic tubular structure (TC). During the procedure, the operator steadily slides the transducer cephalad from the suprasternal notch until it cannot be moved further, which would be completed within 30 s.

All investigators (CHW, CYW, MCW, JT) performing the imaging acquisition procedure had at least 10 years of experience in point-of-care ultrasound use in the emergency department and received training to standardize the image acquisition procedure before the study’s inception. Three investigators (CYW, MCW, JT) collected imaging data at XXXH and the other (CHW) at XXXH-YB. The ultrasound machines used for the study were Xario 100 (Canon Medical Systems Corporation, Ōtawara, Tochigi, Japan) (frame rate: 30 frames per second, image size: 960 × 720 pixels) at XXXH and LOGIQ e (GE Healthcare, Chicago, IL) (frame rate: 30 frames per second, 800 × 600 pixels) at XXXH-YB. The ultrasound images were acquired with a 12L-RS high-frequency linear transducer.

### Image Labelling and Ground Truth

Annotation was conducted frame by frame for each video clip. All frames were manually annotated using bounding boxes to mark pixels belonging to CC or TC. The bounding boxes were intended to contain the target pixels with the minimum rectangle areas. Each video clip was randomly assigned to any two of the investigators (CYW, MCW, JT) for annotation. For each cartilage in each frame, the parameters of the two annotated bounding boxes were averaged to create the ground-truth bounding box for model development.

### Development of the Algorithm

Imaging data were randomly divided into training (70%), validation (15%), and testing (15%) datasets; approximately 40% of the total imaging data came from the participants at XXXH and 60% from those at XXXH-YB. Dataset splitting was performed to ensure that the proportions of participants from XXXH and XXXH-YB were similar across different datasets and that there was no overlap of participants or frames among the datasets.

You Only Look Once (YOLOv5s) [14] was selected as the model architecture for its balance of model performance and efficiency. During the revision process, Faster Regions with Convolutional Neural Network features (Faster R-CNN) [15] and Single Shot Detector (SSD) [16] were also recommended by the reviewers to be tested in this study.

In the training dataset, the default values of the hyperparameters of these algorithms were used for training. The backbones for Faster R-CNN and SSD were ResNet50 [17] and VGG16 [18], respectively. The training model was initialized from a COCO dataset [19] for YOLOv5s and ImageNet [20] for ResNet50 and VGG16. The batch size was 8, 16, and 64, and the learning rate was 0.032, 0.002, and 0.0002 for YOLOv5s, Faster R-CNN, and SSD. A stochastic gradient descent optimizer was used. Binary cross-entropy (BCE) loss and intersection-over-union (IOU) loss supervised the learning process for YOLOv5s; RPN loss (BCE loss and smooth L1 loss) plus RCNN loss (BCE loss and smooth L1 loss) for Faster R-CNN; BCE loss and smooth L1 loss for SSD. The training procedure was stopped when it reached 50 epochs. The best weightings in the validation dataset were used for later model prediction. In each frame, the predicted bounding box of CC or TC would be output only if its prediction probability was (1) highest among all the predicted bounding boxes and (2) above the predetermined output threshold probability. An Intel Xeon 4-core CPU E5-1620 v2 and a Nvidia RTX 2080Ti card with 11 GB memory were used in this study.

### Evaluation Metrics of the Algorithm

We evaluated the performance of the derived algorithms in two stages. In the first stage, we evaluated whether the model could correctly indicate the presence or absence of CC or TC in the frame, regardless of the location. The metrics of this classification performance included sensitivity, specificity, positive predictive value (PPV), negative predictive value, accuracy, F1-score, and the area under the receiver operating characteristic (ROC) curve (AUC). The optimal output threshold probability was determined by Youden’s index when the derived algorithms were tested on the training dataset. In the second stage, we assessed how accurately the model could indicate the location of CC or TC. The metrics of this localization performance were represented by the IOU. For any given two areas, the IOU was computed as the intersection of the two areas divided by the union of the same areas. IOU was only computed among those frames classified as true-positive (TP) in the first stage and, therefore, was termed the TP-IOU. TP-IOU was calculated to evaluate not only the consistency between the predicted and ground-truth bounding boxes but also between the two manually annotated bounding boxes.

### Statistical Analysis

For descriptive statistics, categorical variables are presented as counts with proportions, and continuous variables are presented as means with standard deviations (SDs). We first calculated the evaluation metrics for each participant according to the participant-specific frames. Subsequently, we calculated the mean value by averaging these participant-specific evaluation metrics and obtained a 95% confidence interval (CI) by a bootstrap technique with 1000 repetitions. Sensitivity, specificity, PPV, negative predictive value, accuracy, F1-score, and TP-IOU were compared by Friedman test. If the Friedman test revealed significant between-group differences, post-hoc pair-wise comparison was conducted by Wilcoxon signed-rank test. The pair-wise comparison in AUC was performed by the DeLong test of correlated ROC curves [21]. Also, the Wilcoxon signed rank test was performed to compare TP-IOU of predicted and ground-truth bounding boxes with the TP-IOU of the two annotated bounding boxes in each frame. The kappa coefficient was calculated to assess the inter-annotator agreement in classifying the presence of CC or TC. The comparisons were also presented in subgroup analysis stratified by XXXH and XXXH-YB. A two-tailed *p*-value < 0.05 was considered statistically significant.

## Results

A total of 488 participants were enrolled in the study, including 205 from XXXH and 283 from XXXH-YB. Their mean age was 36.0 years (SD: 9.0 years), and their mean body mass index was 22.6 (SD: 6.2) kg/m^2^. These participants contributed to a total of 292,053 frames, which were further separated into training (205,931 frames), validation (44,851 frames), and testing datasets (41,271 frames). The splitting process of the dataset is presented in Table 1.

**Table 1:** Baseline characteristics of the included participants stratified by different datasets

	XXXH			XXXH-YB		
	Training	Validation	Testing	Training	Validation	Testing
Age, year	33.7 (8.0)	37.1 (8.3)	38.0 (14.2)	36.6 (8.4)	36.8 (10.4)	37.5 (8.0)
Body mass index, kg/m^2^	21.7 (3.5)	21.8 (3.4)	22.4 (3.2)	22.7 (3.6)	22.3 (3.5)	26.3 (17.8)
Patient number, *n*	143 (29.3)	31 (6.4)	31 (6.4)	198 (40.6)	44 (9.0)	41 (8.4)
Video frame number, *n*	37,346 (12.8)	7075 (2.4)	6961 (2.4)	168,585 (57.7)	37,776 (12.9)	34,310 (11.7)

Data are presented as mean (standard deviation) or count (proportion)

**Fig. 1:**
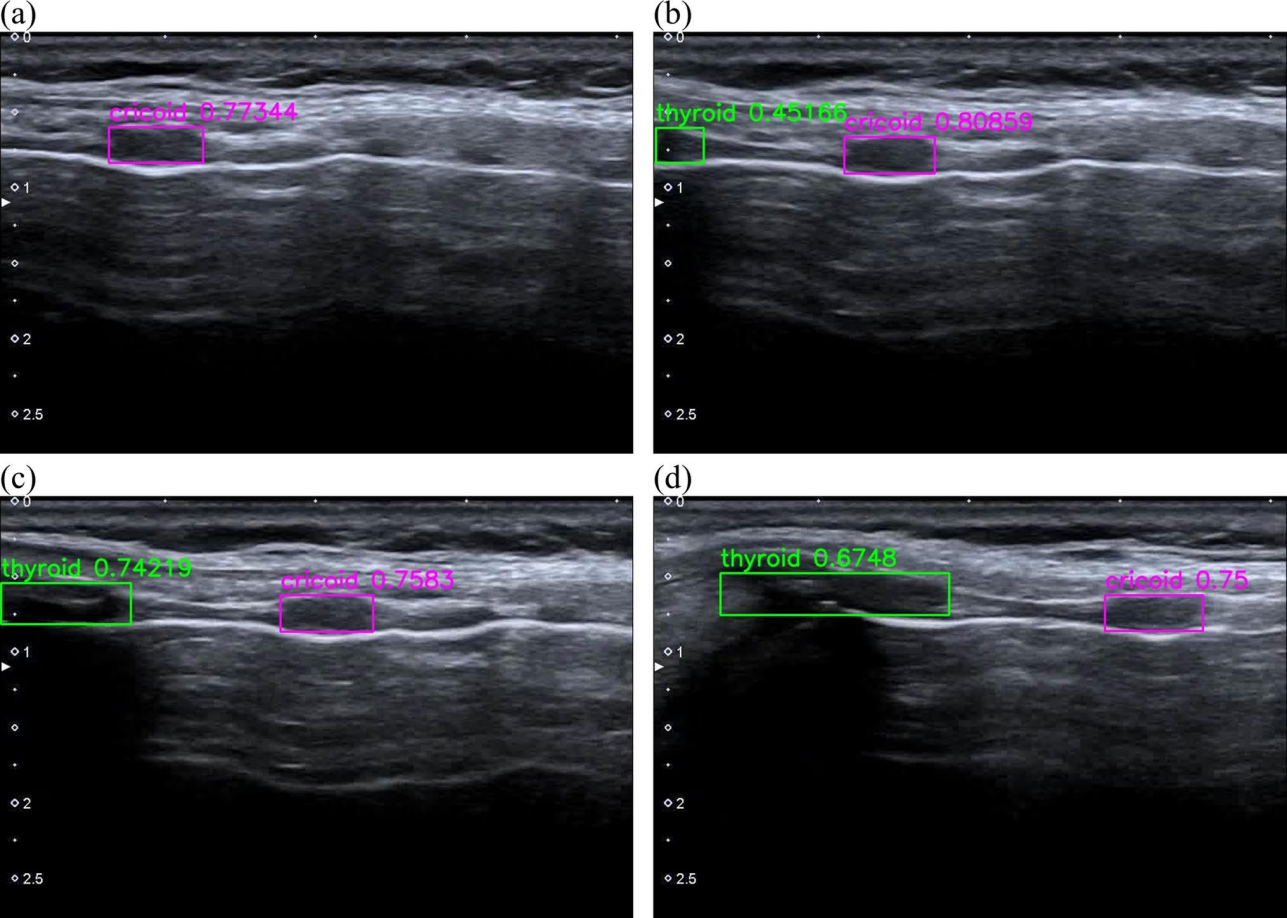
Representative frames demonstrating how YOLOv5s CC/TC prediction model outputs the predicted bounding boxes. The complete video clip can be watched in Video A.2. The image sequence from (**a**) to (**d**) represents the images the sonographers see when they move the transducer from the suprasternal notch cephalad. The pink rectangles indicate the predicted bounding box of the cricoid cartilage and the green rectangles refer to the predicted bounding box of the thyroid cartilage. The output probabilities of the predicted bounding boxes are annotated along with the predicted bounding boxes. Please refer to Video A.2 for the complete video clip

**Fig. 2:**
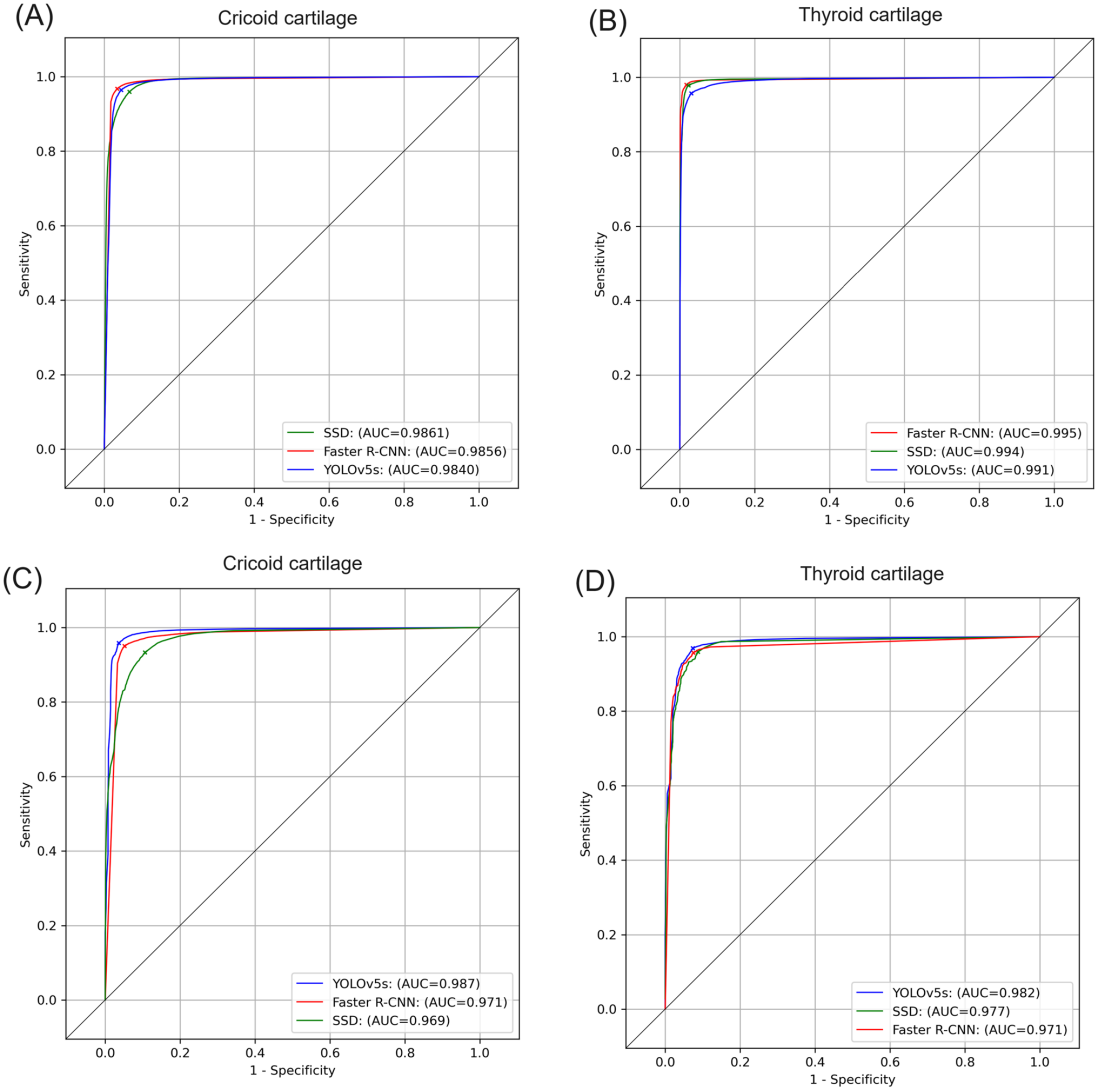
ROC curves. **A**, **B** ROC curves for the derived algorithms in the training datasets; **C**, **D** ROC curves for the derived algorithms in the testing datasets. The output thresholds of the algorithms were determined by Youden’s index in the testing dataset and marked as ticks on the ROC curves (A) and (B). The output thresholds of cricoid cartilage were 0.46, 0.999, and 0.43 for YOLOv5s, Faster R-CNN, and SSD. The output thresholds of thyroid cartilage were 0.39, 0.96, and 0.20 for YOLOv5s, Faster R-CNN, and SSD. ROC, receiver operating characteristic; AUC, area under the ROC curve

In the training dataset, as shown in Fig. 1 and Video A.2, the derived algorithms would output the bounding box for CC or TC when its prediction probability was highest among all predicted bounding boxes and above a certain output threshold. According to the ROC curves (Fig. 2), the thresholds for detecting CC and TC were determined.

In the testing dataset, all three algorithms achieved excellent classification performance for both CC and TC, demonstrating high sensitivity and PPV (Table 2). There were no between-group differences in discriminative performance regarding AUC. Furthermore, in the frames where the model correctly indicated the presence of CC or TC, the algorithms also accurately indicated the location of CC (TP-IOU: YOLOv5s, 0.753, 95% CI: 0.739–0.765; Faster R-CNN, 0.720, 95% CI: 0.709–0.732; SSD, 0.739, 95% CI: 0.726–0.751) and TC (TP-IOU: YOLOv5s, 0.739, 95% CI: 0.722–0.755; Faster R-CNN, 0.709, 95% CI: 0.687–0.730; SSD, 0.713, 95% CI: 0.695–0.730). For both CC and TC, the TP-IOU values were significantly higher in YOLOv5s than Faster R-CNN or SSD. During model testing, YOLOv5s, Faster R-CNN, and SSD could output mean frames per second (FPS) of 62.8 (SD: 0.6), 5.3 (SD: 0.04), and 10.5 (SD: 0.06), respectively.

**Table 2:** Performance of the CC/TC prediction model on the testing dataset

Parameters	Cartilage	Overall comparison, *p*-value	YOLOv5s	YOLOv5s vs. faster R-CNN, *p*-value	Faster R-CNN	Faster R-CNN vs. SSD, *p*-value	SSD	YOLOV5s vs. SSD, *p*-value
***Total***								
Sensitivity	CC	< 0.001	0.956 (0.940, 0.970)	0.006	0.939 (0.913, 0.961)	< 0.001	0.880 (0.851, 0.909)	< 0.001
	TC	0.002	0.937 (0.907, 0.964)	0.57	0.930 (0.893, 0.957)	0.004	0.919 (0.899, 0.939)	0.003
Specificity	CC	< 0.001	0.965 (0.918, 0.992)	0.074	0.955 (0.901, 0.987)	0.001	0.932 (0.901, 0.957)	< 0.001
	TC	< 0.001	0.947 (0.895, 0.982)	0.8	0.944 (0.899, 0.981)	0.004	0.943 (0.891, 0.980)	0.063
PPV	CC	< 0.001	0.993 (0.983, 0.998)	0.113	0.990 (0.980, 0.997)	< 0.001	0.983 (0.976, 0.989)	< 0.001
	TC	< 0.001	0.990 (0.982, 0.996)	0.572	0.991 (0.984, 0.996)	< 0.001	0.987 (0.979, 0.992)	0.047
NPV	CC	< 0.001	0.728 (0.634, 0.815)	0.049	0.679 (0.583, 0.772)	< 0.001	0.521 (0.436, 0.615)	< 0.001
	TC	0.01	0.843 (0.784, 0.894)	0.353	0.824 (0.763, 0.879)	0.017	0.748 (0.680, 0.814)	0.004
Accuracy	CC	< 0.001	0.960 (0.946, 0.973)	< 0.001	0.944 (0.918, 0.964)	< 0.001	0.895 (0.868, 0.918)	< 0.001
	TC	< 0.001	0.946 (0.921, 0.966)	0.789	0.940 (0.911, 0.963)	< 0.001	0.930 (0.914, 0.946)	< 0.001
F1 score	CC	< 0.001	0.972 (0.962, 0.981)	< 0.001	0.959 (0.937, 0.975)	< 0.001	0.923 (0.904, 0.941)	< 0.001
	TC	< 0.001	0.956 (0.931, 0.974)	0.788	0.950 (0.917, 0.974)	< 0.001	0.949 (0.936, 0.961)	< 0.001
AUC	CC	NA	0.989 (0.982, 0.994)	0.10	0.986 (0.980, 0.991)	0.06	0.968 (0.956, 0.977)	0.12
	TC	NA	0.989 (0.977, 0.997)	0.10	0.981 (0.965, 0.991)	0.13	0.982 (0.973, 0.990)	0.12
TP-IOU	CC	< 0.001	0.753 (0.739, 0.765)	< 0.001	0.720 (0.709, 0.732)	< 0.001	0.739 (0.726, 0.751)	0.003
	TC	< 0.001	0.739 (0.722, 0.755)	< 0.001	0.709 (0.687, 0.730)	0.668	0.713 (0.695, 0.730)	< 0.001
***XXX hospital***								
Sensitivity	CC	< 0.001	0.988 (0.978, 0.997)	0.01	0.960 (0.909, 0.991)	0.004	0.912 (0.859, 0.954)	< 0.001
	TC	< 0.001	0.973 (0.956, 0.988)	0.06	0.950 (0.914, 0.977)	0.015	0.926 (0.896, 0.951)	< 0.001
Specificity	CC	0.867	0.698 (0.095, 1.000)	NA	0.667 (0.000, 1.000)	NA	0.792 (0.476, 1.000)	NA
	TC	0.325	0.868 (0.751, 0.965)	NA	0.869 (0.755, 0.960)	NA	0.878 (0.762, 0.975)	NA
PPV	CC	0.867	0.991 (0.974, 1.000)	NA	0.991 (0.972, 1.000)	NA	0.994 (0.982, 1.000)	NA
	TC	0.223	0.982 (0.963, 0.994)	NA	0.987 (0.971, 0.997)	NA	0.985 (0.967, 0.996)	NA
NPV	CC	0.135	0.234 (0.065, 0.494)	NA	0.085 (0.000, 0.208)	NA	0.069 (0.000, 0.173)	NA
	TC	0.023	0.798 (0.680, 0.902)	0.041	0.745 (0.612, 0.860)	0.082	0.604 (0.481, 0.722)	0.005
Accuracy	CC	< 0.001	0.979 (0.959, 0.995)	0.006	0.951 (0.891, 0.988)	0.016	0.908 (0.859, 0.948)	< 0.001
	TC	0.008	0.961 (0.941, 0.979)	0.42	0.947 (0.917, 0.970)	0.009	0.923 (0.895, 0.947)	0.001
F1 score	CC	< 0.001	0.989 (0.976, 0.997)	0.007	0.967 (0.925, 0.994)	0.015	0.945 (0.911, 0.971)	< 0.001
	TC	0.003	0.976 (0.963, 0.986)	0.416	0.965 (0.943, 0.981)	0.01	0.951 (0.933, 0.966)	0.001
AUC	CC	NA	0.963 (0.889, 1.000)	0.27	0.973 (0.918, 1.000)	0.03	0.951 (0.882, 1.000)	0.07
	TC	NA	0.979 (0.947, 0.997)	0.20	0.982 (0.965, 0.994)	0.16	0.971 (0.951, 0.987)	0.19
TP-IOU	CC	< 0.001	0.744 (0.720, 0.765)	0.002	0.711 (0.688, 0.731)	0.015	0.738 (0.717, 0.761)	0.692
	TC	0.031	0.750 (0.726, 0.773)	0.025	0.733 (0.709, 0.758)	0.992	0.732 (0.705, 0.755)	0.008
***XXX hospital—Yunlin branch***								
Sensitivity	CC	< 0.001	0.932 (0.906, 0.953)	0.135	0.924 (0.895, 0.948)	0.001	0.856 (0.818, 0.891)	< 0.001
	TC	0.102	0.910 (0.857, 0.951)	NA	0.914 (0.861, 0.958)	NA	0.914 (0.885, 0.940)	NA
Specificity	CC	< 0.001	0.984 (0.972, 0.993)	0.117	0.977 (0.960, 0.989)	< 0.001	0.942 (0.920, 0.961)	< 0.001
	TC	< 0.001	0.997 (0.994, 0.999)	0.108	0.993 (0.984, 0.998)	< 0.001	0.984 (0.972, 0.992)	< 0.001
PPV	CC	< 0.001	0.993 (0.988, 0.998)	0.166	0.990 (0.982, 0.996)	< 0.001	0.975 (0.968, 0.983)	< 0.001
	TC	< 0.001	0.996 (0.993, 0.999)	0.295	0.994 (0.990, 0.998)	< 0.001	0.988 (0.981, 0.994)	< 0.001
NPV	CC	< 0.001	0.861 (0.811, 0.904)	0.117	0.853 (0.801, 0.894)	< 0.001	0.752 (0.698, 0.796)	< 0.001
	TC	0.068	0.874 (0.813, 0.926)	NA	0.878 (0.821, 0.925)	NA	0.857 (0.807, 0.901)	NA
Accuracy	CC	< 0.001	0.946 (0.923, 0.962)	0.034	0.938 (0.916, 0.957)	< 0.001	0.884 (0.860, 0.908)	< 0.001
	TC	0.016	0.934 (0.890, 0.966)	0.752	0.935 (0.889, 0.969)	0.023	0.936 (0.913, 0.956)	0.074
F1 score	CC	< 0.001	0.960 (0.944, 0.972)	0.03	0.953 (0.933, 0.968)	< 0.001	0.907 (0.882, 0.928)	< 0.001
	TC	0.016	0.941 (0.899, 0.972)	0.783	0.939 (0.887, 0.974)	0.027	0.947 (0.927, 0.963)	0.078
AUC	CC	NA	0.991 (0.986, 0.995)	0.09	0.987 (0.981, 0.992)	0.06	0.969 (0.959, 0.979)	0.12
	TC	NA	0.995 (0.990, 0.999)	0.04	0.980 (0.957, 0.994)	0.11	0.990 (0.985, 0.994)	0.08
TP-IOU	CC	< 0.001	0.760 (0.746, 0.774)	< 0.001	0.727 (0.715, 0.739)	0.008	0.740 (0.725, 0.754)	< 0.001
	TC	< 0.001	0.730 (0.707, 0.754)	< 0.001	0.690 (0.657, 0.721)	0.564	0.698 (0.671, 0.722)	< 0.001

The metrics are expressed with point estimates and corresponding 95% confidence intervals. *AUC* area under the receiver operating characteristic curve, *CC* cricoid cartilage, *NA* not available, *NPV* negative predictive value, *PPV* positive predictive value, *TC* thyroid cartilage *TP-IOU* true positive-intersection over union

Compared with the TP-IOU values of predicted and ground-truth bounding boxes, the TP-IOU value of the two annotated bounding boxes was significantly lower for CC, while comparable for TC (Table 3). The kappa values indicated high inter-annotator agreement for both CC and TC regarding the judgment of the presence or absence of the cartilages.

**Table 3:** Comparison of TP-IOU between model predictions and manual annotations

Target cartilage	TP-IOU							Kappa coefficient
	YOLOv5s	*p-*value	Faster R-CNN	*p-*value	SSD	*p-*value	Annotators	annotators
*Total*								
CC	0.753 (0.739, 0.765)	< 0.001	0.72 (0.709, 0.732)	0.004	0.739 (0.727, 0.752)	< 0.001	0.684 (0.661, 0.705)	0.915 (0.859, 0.962)
TC	0.739 (0.722, 0.755)	0.584	0.709 (0.688, 0.728)	0.265	0.713 (0.694, 0.73)	0.235	0.733 (0.708, 0.758)	0.901 (0.847, 0.949)
*XXX hospital*								
CC	0.744 (0.719, 0.766)	< 0.001	0.711 (0.688, 0.731)	0.003	0.738 (0.715, 0.758)	< 0.001	0.672 (0.647, 0.695)	0.868 (0.740, 0.967)
TC	0.75 (0.726, 0.772)	0.977	0.733 (0.71, 0.758)	0.135	0.732 (0.708, 0.758)	0.318	0.750 (0.714, 0.782)	0.827 (0.707, 0.919)
*XXX hospital-Yunlin branch*								
CC	0.76 (0.746, 0.773)	< 0.001	0.727 (0.715, 0.738)	0.098	0.74 (0.727, 0.753)	0.008	0.693 (0.659, 0.723)	0.950 (0.923, 0.972)
TC	0.73 (0.707, 0.754)	0.720	0.69 (0.659, 0.72)	0.309	0.698 (0.671, 0.724)	0.309	0.721 (0.685, 0.757)	0.957 (0.916, 0.983)

*CC* cricoid cartilage, *TC* thyroid cartilage, *TP-IOU* true positive-intersection over union. The metrics are expressed with point estimates and corresponding 95% confidence intervals. The *p-*value indicates the comparison between each algorithm and annotator

In subgroup analysis, the model performance and the agreement evaluation were similar between XXXH and XXXH-YB (Tables 2 and 3).

## Discussion

The ML-based algorithms correctly identified anatomical landmarks for cricothyroidotomy on sagittal ultrasound images in adult females. By analyzing 292,053 frames collected from 488 participants, the derived YOLOv5s, Faster R-CNN, and SSD algorithms recognized the CC and TC with high sensitivity and accurate localization.

### Comparison with Previous Studies

Regarding cricothyroidotomy, few studies [10, 22] are focused only on women. In one prospective study, 24 physicians were able to correctly identify the CTM by manual palpation in only 13 (23%) of 56 women [23]. Interestingly, most of the physicians rated the palpation difficulty level as easy [23]. This suggests that many of the physicians thought they had correctly identified the CTM when they may have actually misidentified it [23].

The Difficult Airway Society guidelines have proposed ultrasonography to assist in identifying the CTM [7]. However, Hung et al., in a meta-analysis [10], have reported that although the pooled failure rate of the ultrasound-guided technique was significantly lower than that of manual palpation (300/535, 56%), it was nonetheless as high as 26% (147/559). Because the analysis by Hung et al. [10] included a mixture of study participants with a high level of clinical heterogeneity, there remained a paucity of evidence supporting the application of ultrasonography in identifying the CTM in women.

### Interpretation of Current Results

To the best of our knowledge [10, 22], our study has enrolled the largest group of adult female participants to date in investigating ultrasonography for identifying anatomic landmarks of cricothyroidotomy in women. Two ultrasound techniques, transverse and longitudinal, have been advocated to guide cricothyroidotomy [24]. In the transverse technique, the sonographer moves the transducer back and forth around the CTM (i.e., between the TC and CC). Our ML-based algorithms were intended for clinicians who may not be well versed in sonographic techniques and, therefore, may not have the a priori knowledge necessary for recognizing CC or TC, which is a prerequisite for the transverse technique. On the other hand, the longitudinal technique has no such prerequisite and may be more suitable for those clinicians with less exposure to neck sonoanatomy. To facilitate the application of the ML-based algorithms, we further simplified the longitudinal technique by allowing the clinicians to use the suprasternal notch as a landmark, making it the starting point, and moving the transducer cephalad along the sagittal midline. This modified technique did not demand prior knowledge of either transverse or longitudinal techniques and may thus be more favorable for novice sonographers. Finally, instead of the CTM per se, we chose the CC and TC as the target objects to train the algorithm. These two cartilages are also the critical landmarks used by the conventional transverse and longitudinal techniques [24]. We assumed that localizing CC and TC by the derived ML-based algorithms would be sufficient to identify the CTM.

In our study, detection-based algorithms, including YOLOv5s, Faster R-CNN, and SSD, were adopted as the model architecture. For these algorithms, the output threshold probability determined the number and accuracy of the predicted bounding boxes. In the default settings, the trained algorithms would output all predicted bounding boxes if their predicted probabilities were above a certain threshold. Therefore, the trained algorithms could have output more than one bounding box for CC or TC in a single frame, as long as the predicted probabilities of the bounding boxes were above the threshold. Because an excess of predicted bounding boxes might distract the sonographer from identifying the CTM in clinical practice, we decided that only the bounding box with the highest predicted probability among boxes with probabilities above the threshold would be output. Subsequently, according to the ROC curve (Fig. 2), Youden’s index was adopted to find the optimal cut-off, striking a balance between the sensitivity and specificity, and this threshold was thereby used in the testing datasets.

We used two steps to evaluate the classification and localization performance of the derived algorithms (Table 2). We assumed this staged assessment would make the model’s performance more easily understood by clinicians. In the first step, we used classification metrics, most importantly, sensitivity and PPV, to determine how correctly the model could identify the presence of CC or TC. Since cricothyroidotomy is a time-sensitive procedure, the model should be very sensitive to the presence of CC or TC. In the meantime, it should also maintain a sufficiently high PPV to avoid an excessive number of false alarms and subsequent sonographer attention fatigue. As shown in Table 2, the PPV of the three derived algorithms were all above 0.98 for both CC and TC, demonstrating excellent classification performance of these algorithms.

However, simply indicating the presence of the cartilages in the frame may not be enough to help clinicians identify the position of the CTM. To correctly position the CTM, the predicted bounding boxes should have as much overlap with the cartilages as possible. Therefore, in the second stage, IOU was adopted to express the level of this overlap. After the first stage, the frames would be classified into four categories, true-positive, false-negative, false-positive, and true-negative, based on the presence or absence of predicted and ground-truth bounding boxes. For the frames that were incorrectly classified (false negative or false positive) at the first stage, the IOU was zero, which offers no more information than the classification result. For those frames correctly classified as true negative, the IOU could not be calculated. Therefore, only the IOU of the true-positive frames (TP-IOU) is considered in localizing the membrane, and clinicians can know how much overlap there was in order to understand how accurately the predicted bounding boxes had localized the cartilages.

### Future Applications

While ultrasound has generally been accepted as more accurate than manual palpation in identifying TC or CC in clinical trials, it can also require significantly more time than manual palpation for initial identification of the CTM and insertion of the airway device [25–27]. This ultrasound-related time lag may be caused by clinicians’ unfamiliarity with the airway sonoanatomy [28]. As such, the likelihood is high that clinicians may forget how to identify TC or CC by ultrasound when they are faced with an emergent need like cricothyroidotomy. Our ML-based algorithms may solve this dilemma by offering a real-time guide for those less-experienced sonographers. Also, as shown in Table 2, the accuracy of the ML-based algorithms was not influenced by different ultrasound machines of different hospitals with different image resolutions or image quality, indicating its potential to be generalizable. Integration of the ML-based algorithms with the hand-held portable ultrasound should be further explored for its potential in real-time guidance for cricothyroidotomy. Given the higher TP-IOU and FPS, YOLOv5s may be considered the optimal algorithm to be deployed in portable ultrasound machines.

### Study Limitations

First, we only enrolled female participants without obvious neck deformity. Therefore, whether the ML-based algorithms could be applied to males or patients with neck deformities should be further explored. Second, as with the original longitudinal technique, patients with a short neck may not be easily approached with our modified longitudinal technique, rendering the ML-based algorithms inapplicable. Third, the mean body mass index was 22.6 kg/m^2^ in our study. External generalization of the current algorithms to participants with elevated body mass index or morbid obesity may not be applicable. Fourth, the ML-based algorithms were not tested in an external dataset. However, no publicly available datasets of ultrasonographic images of CC/TC could be used for testing. In the current study, the images were collected by four physicians from two hospitals using two different ultrasound machines. The model performance was not significantly different between these two hospitals, which may suggest that the external generalization of the derived algorithms may be favorable. Finally, the evaluation metrics were solely based on image analysis. It is still unknown whether the favorable metrics of the ML-based algorithms could be beneficial clinically. To test the derived algorithms clinically, the algorithms should be transferred to hand-held portable ultrasound devices since it is less likely that the ML-based algorithms would be used in conventional cart-based ultrasound machines. Notwithstanding, either would involve much technical work before the trained algorithms could be widely tested in the clinical setting.

## Conclusions

The ML-based algorithms, including YOLOv5s, Faster R-CNN, and SSD, identified anatomical landmarks for cricothyroidotomy in adult females with high sensitivity and accurate localization via ultrasonographic images. Different ultrasound machines did not influence the performance of the derived algorithms. Given the higher TP-IOU and FPS, YOLOv5s may be considered the optimal algorithm for clinical use in portable ultrasound machines.

## Appendices

**     Video ****A.1.** A video clip to demonstrate the modified longitudinal technique for acquiring the sonographic imaging data used in the study.

**Video ****A.2.** A video clip to demonstrate the predictions output by the YOLOv5s CC/TC prediction model. Pink rectangles indicate the predicted bounding box of the cricoid cartilage and green rectangles refer to the predicted bounding box of the thyroid cartilage. The output probabilities of the predicted bounding boxes are annotated along with the predicted bounding boxes.

**Supplemental ****Fig. 1.** The architecture of the YOLOv5s network. Each ultrasonographic video clip is first sliced into an image sequence, and each image is resized to 640 × 640 pixels before input. A convolutional neural network (Backbone) is used to extract features of the input image in different granularities. Extracted feature maps are further mixed and up-sampled in the Head. Finally, in Detect, the detection results (bounding box position and probability of each class) are output in three different scales.

**Supplemental ****Fig. 2.** The architecture of the Faster R-CNN unified network. Each ultrasonographic video clip is first sliced into an image sequence, and each image is resized to 640 × 640 pixels before input. A feature extractor, ResNet-50, was taken as a backbone to extract input image features and share them with the region proposal network (RPN). RPN-predicted anchor boxes allow the unified network to attend the region of interest (ROI) on the feature map and thus crop the ROI and feed it into the classifier (red rectangle) to complete the prediction.

**Supplemental ****Fig. 3.** The architecture of the SSD network. Each ultrasonographic video clip is first sliced into an image sequence, and each image is resized to 300 × 300 to fit the SSD300 lightweight version. A base network, VGG-16, was the backbone to extract input image features. For each location on the feature map, the following feature layers directly predict a bounding box shape offset, scores for every category, in different resolutions layer by layer. A non-maximum suppression algorithm is applied before output.

### Supplementary Information

Below is the link to the electronic supplementary material.

Supplementary file1 (TIF 614 KB)

Supplementary file2 (TIF 200 KB)

Supplementary file3 (TIF 224 KB)

Supplementary file4 (MP4 4417 KB)

Supplementary file5 (MP4 1313 KB)

## Data Availability

The datasets generated during and/or analyzed during the current study are available from the corresponding author on reasonable request.
