# Supplementary figures and images for: Application of Machine Learning to Ultrasonography in Identifying Anatomical Landmarks for Cricothyroidotomy Among Female Adults: A Multi-center Prospective Observational Study
Source: J Imaging Inform Med. 2024 Jan 10;37(1):363–73. doi: 10.1007/s10278-023-00929-3 (PMC11031510; doi:10.1007/s10278-023-00929-3)

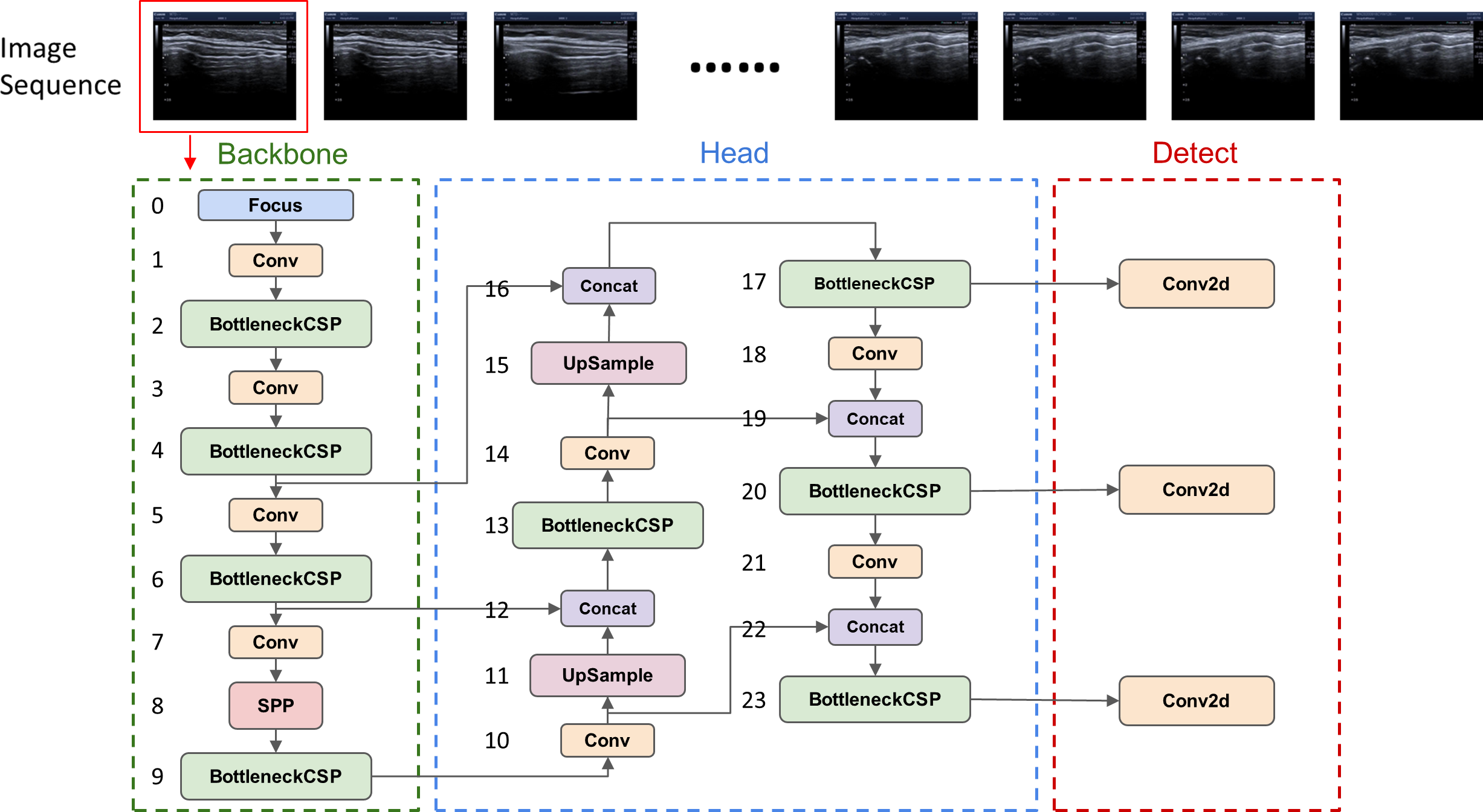

Supplement: Supplementary file 1 — Supplementary file1 (TIF 614 KB) [file 10278_2023_929_MOESM1_ESM.tif]

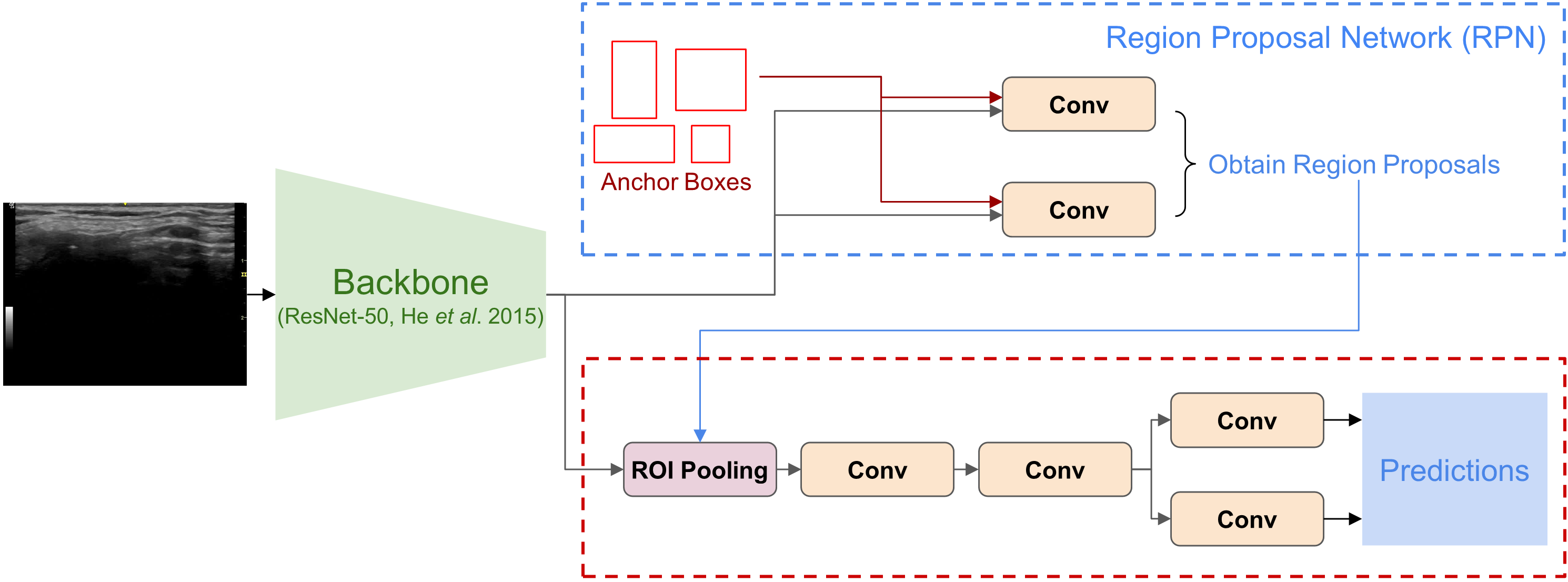

Supplement: Supplementary file 2 — Supplementary file2 (TIF 200 KB) [file 10278_2023_929_MOESM2_ESM.tif]

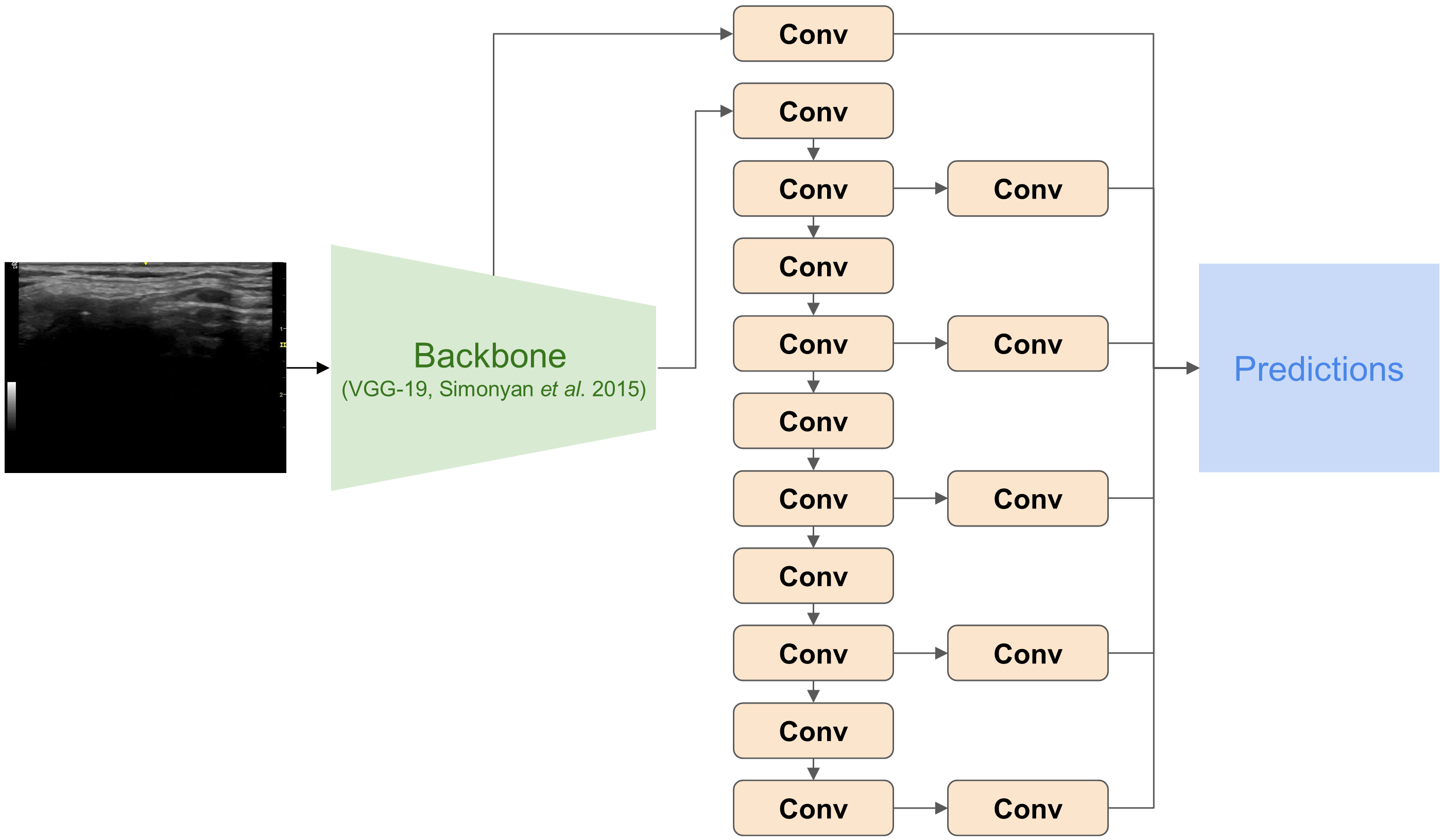

Supplement: Supplementary file 3 — Supplementary file3 (TIF 224 KB) [file 10278_2023_929_MOESM3_ESM.tif]
